# Getting the Message Out: the Many Modes of Host-Symbiont Communication during Early-Stage Establishment of the Squid-Vibrio Partnership

**DOI:** 10.1128/mSystems.00867-21

**Published:** 2021-09-28

**Authors:** Margaret McFall-Ngai, Edward Ruby

**Affiliations:** a Kewalo Marine Laboratory, Pacific Biosciences Research Center, School of Ocean and Earth Science and Technology, University of Hawaiʻi at Mānoa, Honolulu, Hawaii, USA; University of California San Diego

**Keywords:** horizontal transmission, microbiome, development, signal/cue, animal development

## Abstract

Symbiosis, by its basic nature, depends on partner interactions that are mediated by cues and signals. This kind of critical reciprocal communication shapes the trajectory of host-microbe associations from their onset through their maturation and is typically mediated by both biochemical and biomechanical influences. Symbiotic partnerships often involve communities composed of dozens to hundreds of microbial species, for which resolving the precise nature of these partner interactions is highly challenging. Naturally occurring binary associations, such as those between certain legumes, nematodes, fishes, and squids, and their specific bacterial partner species offer the opportunity to examine interactions with high resolution and at the scale at which the interactions occur. The goals of this review are to provide the conceptual framework for evolutionarily conserved drivers of host-symbiont communication in animal associations and to offer a window into some mechanisms of this phenomenon as discovered through the study of the squid-vibrio model. The discussion focuses upon the early events that lead to persistence of the symbiotic partnership. The biophysical and biochemical determinants of the initial hours of dialogue between partners and how the symbiosis is shaped by the environment that is created by their reciprocal interactions are key topics that have been difficult to approach in more complex systems. Through our research on the squid-vibrio system, we provide insight into the intricate temporal and spatial complexity that underlies the molecular and cellular events mediating successful microbial colonization of the host animal.

## INTRODUCTION

## INTRODUCTION—THE LEXICON: CUES VERSUS SIGNALS AND HORIZONTAL VERSUS VERTICAL TRANSMISSION

Although this piece centers on the early interactions of host and symbiont in the squid-vibrio symbiosis, we believe that it is important to bring the reader to an understanding of the definitions that we use for certain terms. As such, we start off our discussion here by considering some concepts relevant to the biology of the model that we study.

In animal symbioses, three types of interactions can be distinguished: host-host (important in horizontally transmitted symbioses, such as in the mother-newborn interactions of mammals), microbe-microbe, and host-microbe. In each of these cases, interorganism communication is key and is based on cues and/or signals. Both terms refer to biochemical or biophysical stimuli from one organism that evoke a biological response in an associated organism. The difference is that, in the case of a cue, the selection pressure on the organism emitting the cue was not driven by its ability to elicit a response in the receiving organism. In contrast, signal molecules are produced for the purpose of generating a particular response. Examples of molecules that often act as symbiont-host cues are microbe-associated molecular patterns, or MAMPs, such as the cell envelope constituents of bacteria (e.g., peptidoglycan [PGN] or lipopolysaccharide [LPS]) (1). The shedding of LPS is a shared, derived character of most Gram-negative bacteria, whether they are free-living or host associated; similarly, PGN fragments are released from the cell walls of both Gram-positive and -negative bacteria. The host uses the detection of these passively leaked constituents as a cue to the presence of bacteria. However, MAMP production can evolve to become a signal with a purpose. For example, during growth, bacteria typically recycle their PGN monomer during cell wall remodeling; however, in some species of Gram-negative bacteria, substantial amounts of the PGN monomer are exported, acting as a signal that mediates a specific response in the epithelia of susceptible host species (2).

The mode by which symbionts are transmitted between host generations is also a critical driver of signaling between partners. Here, we use the following terminology. Symbionts are transmitted between generations either (i) vertically, in or on the egg with the microbes incorporated into the events of embryogenesis, or (ii) horizontally, where the symbionts are picked up from the environment anew each generation following a microbe-free embryogenic and/or posthatch period. The nature of symbiosis communication in horizontally transmitted associations between animals and their beneficial bacteria will be the topic of this contribution.

Critical to understanding the nature of horizontal transmission in a given species is a recognition of how the environment shapes the process. This aspect is often not considered, so confusion arises, leading to truly horizontal transmission to be mistaken for vertical transmission. Perhaps the strongest factor in its evolutionary selection is whether the animal host is aquatic or terrestrial. Specifically, in aquatic habitats, microbes of highly coevolved symbioses are shed from host animals into a physically supportive and dispersing aqueous environment. The next generation of juveniles then recruits their specific microbial partners from these reservoir populations, which occur as constituents of the picoplankton (i.e., microbes in the water column). In contrast, host conspecifics often facilitate horizontal transmission of symbionts between generations in terrestrial habitats, which do not typically harbor abundant reservoir populations of the microbial partners ambiently. Thus, evolutionary selection pressure for horizontal symbiont transmission in terrestrial environments has resulted in host-host interactions in some taxa, a level of direct communication that only rarely occurs in aquatic habitats. For example, in mammals, including in terrestrially derived marine mammals, behavioral interactions of the offspring with the mother during birth and nursing are critical for the initial transfer of the mother’s microbiota (3). Another example can be found in termites, where solid excreta of the nestmates is fed to the larvae. Irrespective of the environment in which a symbiosis is horizontally transmitted, the embryonic period, while typically sterile, sets the biophysical and biochemical “stage” for the first interactions with the microbial world at hatching or birth.

## RECRUITMENT TO HOST TISSUES CAN INVOLVE A BIOPHYSICAL AND BIOCHEMICAL “GAUNTLET” THAT SELECTS FOR THE PROPER, COEVOLVED MICROBIAL PARTNER

In the symbiosis between the bobtail squid Euprymna scolopes and the luminous bacterium Vibrio fischeri, the landscape of the light organ surface is the site along which a reciprocal dialogue between host and symbiont mediates the specificity of the symbiosis; i.e., even before the symbionts reach the internal tissues in which they will reside persistently, selection has already begun in tissues ∼100 μm “upstream” of that point.

### Pre- and posthatch events that set the stage for host-symbiont communication.

**FIG 1:**
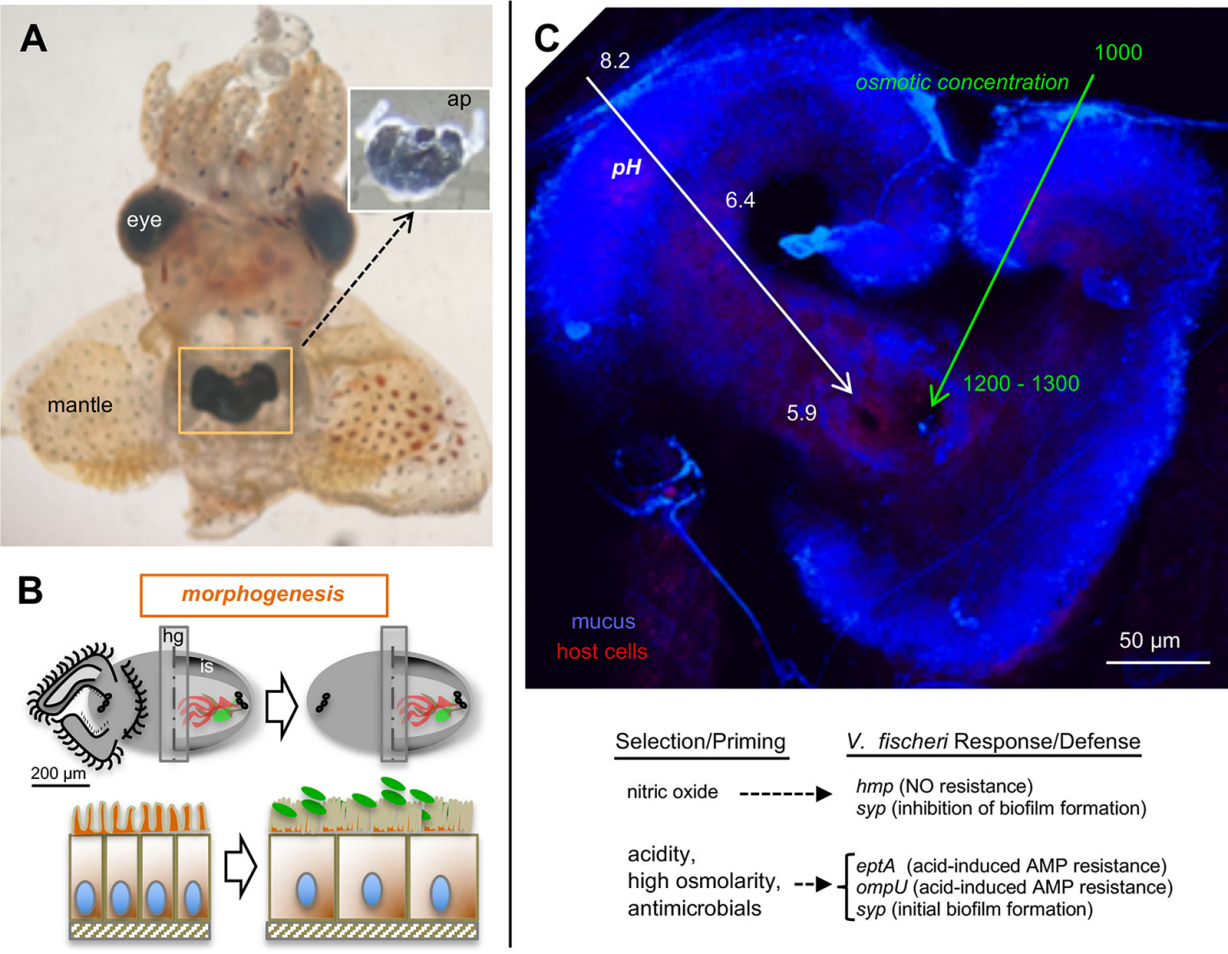
The juvenile light organ of *E. scolopes*. (A) Ventral dissection of a newly hatched animal. The organ, which is embedded in the ink sac, occurs in the center of the mantle cavity. (Upper inset) When the light organ is removed from the body cavity, portions of the transparent ciliated appendages that facilitate colonization can be seen. ap, anterior appendage of one side. (Photo courtesy of L. Hightower.) (B) Symbiont-induced light organ development. (Top) For each diagram, the light organ surfaces at hatching (left) and after ∼96 h (right) are shown. The symbionts induce the loss of these fields. The right side of each diagram shows a cutaway of the organ surface to reveal the positions of the three independent crypts that occur on each side of the organ. (Bottom) The crypts are lined by polarized, microvillous epithelia that undergo changes in response to symbiont colonization. (Left) Columnar epithelia, with sparse microvilli, of the hatchling animal; (right) cuboidal epithelium with dense microvilli of the symbiotic animal. hg, hindgut; is, ink sac. (C) Conditions of symbiont recruitment on the organ surface. (Top) Physical conditions of decreasing pH and increasing osmotic concentration experienced by the environmental reservoir of V. fischeri cells as they transition from their niche as members of the bacterioplankton to becoming symbiont recruits on the organ surface; (bottom) harsh biochemical conditions presented by the host on the nascent organ surface and symbiont genes involved in the molecular responses for adapting to, and exerting dominance in, the locations where symbionts enter and colonize host tissues. AMP, antimicrobial proteins; NO, nitric oxide.

Before outlining host-symbiont interactions, we describe here the landscape in which the dialogue develops in the squid-vibrio partnership (4, 5). As in likely all horizontally transmitted symbioses, during embryogenesis, the host creates a complex nascent symbiotic organ that poises specific animal tissues to recruit the symbiont upon hatching (6). After a 20-day embryonic period, the host (∼2 mm in mantle length) (Fig. 1A) hatches and, within minutes, initiates a cascade of events that will lead to successful colonization of the organ. The anatomical features presented by the hatchling (Fig. 1B) include two ciliated fields on either side of the light organ (7, 8). Each field comprises a region of long, metachronally beating cilia along the outside two apposing appendages and short randomly beating cilia along the inside face of the appendages (9). At the base of each inside face are three pores into which V. fischeri must migrate to colonize internal tissues; only V. fischeri cells persist beyond the pore. Internal features of the nascent organ are also formed during embryogenesis (6). The pores lead to three independent migration paths on each side of the organ, through which the colonizing V. fischeri cells traverse to reach medial, blind-ended crypts. These pathways form sequentially during embryogenesis so that, at hatching, the migratory paths are at different stages of maturity (10, 11). The most mature path begins at the lateral pore and passes along a narrow duct, across a broadened antechamber, and through a very narrow bottleneck that leads to the crypt space. All regions are ciliated and microvillous, except the epithelium-lined crypts, which are principally microvillous (10).

Shortly after the hatching from the egg, two events occur: (i) the migratory pathway of the most mature crypt expands (10), and (ii) the cilia on the organ surface begin to beat, and the associated epithelia shed mucus in a nonspecific response to environmental PGN released by the ambient bacterioplankton (4). The environment in which the symbionts will be enriched is biochemically harsh (Fig. 1C). A pH gradient forms from the seawater environment (pH 8.2), where reservoir populations of V. fischeri reside, through the mucus and to the pores of the light organ (pH 5.9). This journey presents V. fischeri cells with a >100-fold increase in hydrogen ion concentration (12). In addition, the squid tissue is at an osmolarity that is 25% higher than that of seawater. Further, the surface mucus has an array of antimicrobials at low concentrations, including a bactericidal permeability-increasing protein (EsBPI2) (13), a peptidoglycan recognition protein (EsPGRP2) (14), nitric oxide (15), lysozyme (12), cathepsin L (16), a hemocyanin derivative with prophenyloxidase activity (17), and galaxin (18). In addition to containing antimicrobials, the mucus contains polymeric chitin and low levels of an endochitinase that breaks down chitin to its dimeric form, the chemoattractant chitobiose. The activity of this host endochitinase is critical to the eventual colonization by the symbiont (12, 19).

### The process of winnowing.

Nearshore marine habitats typically harbor about a million bacteria/ml of seawater, and in regions where the host is abundant, the cells of various strains of V. fischeri represent <0.1% of the bacterioplankton. Because the host body cavity is only about 1 μl in volume, the water inside functions at low Reynolds’ numbers; i.e., its viscosity is high. Thus, the task of bringing candidate symbionts into the vicinity of the light organ is principally mediated by the activities of the two kinds of cilia on the organ’s surface. The metachronal beat of the long cilia focuses bacterium-sized particles into a stagnant zone directly above the pores and containing the short cilia (9). The random beating of these short cilia is hypothesized to mix the chemoattractants released in this area by the host (12). In the presence of an experimental inoculum of 1,000 to 5,000 V. fischeri cells/ml of seawater, over the course of 3 h, the host aggregates only 5 to 10 of them into regions above the pores on each side of the organ. Other Gram-negative bacteria in the seawater can also aggregate, but when V. fischeri cells are present, they dominate (4). This dominance is not due to growth, as these aggregated bacteria are not growing, but rather, it is due to a competitive dominance by the candidate symbionts at that site. These few V. fischeri cells attach to the cilia on 1 to 2 host epithelial cells near the pores and undergo a period of physiological adjustment, or “priming,” for chemotactic and antimicrobial responses. Notably, the symbionts (i) sense a gradient of chitobiose and induce the chemotactic capability to migrate into the pores (12) and (ii) respond to the low levels of antimicrobial molecules in the aggregate by inducing genes that confer resistance to the even harsher chemical environment (20–22) created by the host further up the migration pathway (23) (Fig. 1).

### Host-symbiont communication on the light organ surface following partner selection.

The host responds to the attachment of the few V. fischeri cells with changes both in cell behavior and in light organ gene expression. Specifically, macrophage-like host cells (hemocytes) migrate into the blood sinuses of the ciliated epithelium (24, 25); interestingly, although not yet fully induced, bacterial mutants unable to produce luminescence are defective in this early phenotype (26). A robust change in gene expression across the ∼10,000 cells of the juvenile light organ, as detected by transcriptome sequencing (RNA-seq) analysis, occurs in response to the 5 to 10 V. fischeri cells aggregating on the light organ surface above the pores (12). The changes in host gene expression include an increase in transcription and translation of genes encoding antimicrobials and the endochitinase. This phenomenon has two effects: the bacteria are primed both for the very high levels of the antimicrobials that they will encounter while migrating through the ducts and for chemotaxis up a chitobiose gradient created at the pores. The attaching bacteria respond to the high levels of NO with expression of a regulatory gene that mediates their detachment from the cilia (27). Once detached, the cells chemotax into the pores.

## A FEW HOURS OF SELECTION AND PRIMING READY THE PARTNERS FOR AN EXCLUSIVE, LONG-TERM PARTNERSHIP

Interactions between the squid host and its bacterial partner lead to the development of a microbiogeography and ambient biochemistry that promote the restriction of symbionts to one location in the body and creates an environment that fosters the function of the symbiosis.

### Developmental events—the first hours.

Symbiont-induced cuing of development, which begins as V. fischeri cells aggregate on the surface, is a complex series of events that occur across a biogeographically elaborate landscape. During the symbiont’s journey to each of the six blind-ended crypts of the light organ, they migrate about 100 μm through a duct and a widened antechamber and into a narrow bottleneck (Fig. 1B), a process that is completed in ∼3 h (10). The bottleneck lumina are so narrow that the bacteria typically move in single file toward the crypt. Interestingly, no matter how many bacteria migrate into the bottlenecks, on average only a single, perhaps the leading, V. fischeri cell enters the crypt space and multiplies to fill this compartment (28, 29).

The symbiont cells then deliver a series of signals that potentiate change in both nearby and remote tissues (30). Developmental changes in the light organ itself include a number of physical and molecular manifestations (Fig. 1B). The phenotypes described below are developmental events observed in the most mature crypt (crypt 1); studies of the less developed crypts (crypts 2 and 3) are under way, with early data indicating that the differences in maturity among crypts affect the triggering of symbiosis-driven phenotypes (10), a pattern that is likely to be a generalized phenomenon during the initiation of other symbioses (31, 32).

**(i) The light organ surface.** MAMPs produced by the symbiont are the principal inducers of cell death in the superficial epithelium; this widespread apoptosis program, which is irreversibly signaled at about 12 h following initial host-symbiont interactions (∼6 h after initial crypt colonization), leads to a 4-day process of tissue regression in the superficial ciliated epithelial fields that potentiates symbiont recruitment. While symbiont MAMPs have been identified as the inducers of this morphogenesis, the signal comes from the V. fischeri population residing in the crypts; how that signal makes its way from there to the surface epithelia remains unknown, but the attenuation of host NO is one event that has been implicated in MAMP-driven morphogenesis. Whatever the mechanism, the loss of cells from the superficial epithelium coincides with a symbiont-induced increase in a matrix metalloproteinase that mediates degradation of the basement membranes supporting the ciliated epithelial cells (33).

**(ii) The symbiont migration path through host tissues.** One outcome of the migration of V. fischeri through host tissues to the crypts is that the pore, duct, and bottleneck constrict. Most dramatically, the bottleneck narrows to ∼2 microns, an effect that retains the dense symbiont population of V. fischeri within the crypt spaces over much of the day [see “(i) Daily rhythms” below]. Symbiont cues or signals that mediate bottleneck closure have not been fully defined, although quorum signaling-associated factors of the symbiont may trigger bottleneck narrowing (10).

**(iii) The crypt epithelia.** Within hours of crypt colonization, the crypt epithelial cells swell, increasing their volume ∼4-fold. This host phenotype does not occur during colonization by V. fischeri cells defective in light production; however, it has not yet been established that perception of the luminescence by the host is the inducer of this change in cell microanatomy. In addition, the symbionts induce an ever-increasing density of the microvilli of the crypt epithelia over the first few days of symbiosis (34), a change in ultrastructure mediated by V. fischeri LPS (35).

### Partner communication promoting persistence.

Once the V. fischeri cells enter the crypts, they proliferate to densities that foster quorum signal-mediated luminescence. The partners then begin behaviors that ensure the continued persistence of the association.

**(i) Daily rhythms.** Beginning the first dawn after hatching, whether colonized or not, the squid host sheds the microvilli on the apical surfaces of its crypt epithelia and releases some 75 to 95% of the crypt contents into the environment (36). If the crypts are colonized, the majority of the symbionts are expelled during the release. This behavior of the organ, while diel, does not appear to be circadian, as its triggering requires a light cue (35, 37). Such a daily expulsion event is made possible by the transient opening of the bottleneck, through which the symbiont population must exit the organ (10, 11). The bacteria continue this cycle of release and regrowth, a behavior that renews the bacterial population.

Evidence for true circadian rhythms in this symbiosis has also been reported. For instance, an increase in symbiont luminescence, which is used by the host in its nocturnal behavior, anticipates the onset of darkness. Studies of this phenomenon suggest that the factor driving the rhythm in luminescence is the host’s control of oxygen delivery to the crypts (37). In addition, in response to cues from the symbiont population, the expression of a clock gene encoding the host cryptochrome, Escry1, cycles in the light organ, peaking with highest symbiont luminescence (38). When colonized by a dark-mutant strain of V. fischeri, Escry1 cycling is not induced; however, the presence of a light cue alone is not sufficient but instead works in synergy with MAMPs to induce the day-night oscillations of this gene’s expression.

**(ii) Reciprocal suppression of host and symbiont immunogenic molecules.** At ∼12 h following colonization, the presentation by V. fischeri of MAMPs in the crypts mediates the irreversible signal for morphogenesis of the ciliated surface that potentiates colonization. However, the accumulation of these constitutively produced and exported LPS and PGN derivatives can be toxic to animal cells. Beginning in the hours after developmental induction, the animal begins to export proteins that break down these MAMPs. These include a peptidoglycan recognition protein with amidase activity (EsPGRP2) that cleaves the PGN monomer (14) and an alkaline phosphatase (EsAP1) that detoxifies the lipid A portion of the LPS by removing its phosphate group (39).

The symbionts also promote the development of a harmonious relationship by attenuating the production of certain host-derived antimicrobials. For example, as mentioned above, symbiont MAMPs depress NO production that, while mediating normal morphogenesis, can also lower bacterial viability, an effect that may compromise the symbiosis (Fig. 1). Thus, the signal/effector functions of NO must be carefully balanced (4). Finally, in a recent report of immune system suppression, a small RNA (sRNA) of the bacterial symbionts (SsrA) enters the host cells and turns down gene expression of RIG-1, an RNA-binding protein that mediates immune responses in animals (40).

The host’s hemocytes also change their behavior toward the symbionts. The hemocytes of nonsymbiotic squid treat V. fischeri cells similarly to other bacteria; i.e., they bind and phagocytose numerous bacterial cells. However, after colonization of the light organ, the hemocytes lose their ability to recognize V. fischeri but retain the capacity to recognize other bacteria at the same rate as they do in the nonsymbiotic host (41). This bacterium-induced change in host hemocyte behavior is mediated, in part, by the symbiont membrane protein OmpU; it has not yet been determined how a mutation in the gene encoding OmpU results in a defect in the ability of V. fischeri cells to evade host hemocytes.

**(iii) Communicating to the rest of the body that a successful symbiosis has been established.** A recent study asked whether responses to V. fischeri colonization were systemic in the host (30). To address this question, the transcriptomes of the gills and eyes, in addition to light organ transcriptomes, were examined in animals in different states of symbiosis. The gills were chosen as they are an immune organ in squids, while the eyes were included as they show a significant molecular, developmental, and morphological convergence with the light organ. Both the eyes and light organ function to modulate light and coordinate to control the counterillumination behavior of the host (4).

The transcriptomes of the organs were analyzed under three conditions: nonsymbiotic conditions or conditions under which the transcriptomes were colonized by wild-type symbionts or symbionts defective in light production (i.e., Δ*lux* mutants). Both eyes and gills responded to colonization by wild-type bacteria with changes in transcription, although little overlap in transcriptional changes occurred across the three organs. The transcriptomic response of the gills was enriched in immune genes. The pattern of transcriptional change in the gills was the same in both wild-type- and Δ*lux* mutant-colonized animals. In contrast, the changes in transcription in the eyes were enriched in genes encoding sensory perception, and the transcriptomic response in the eyes was entirely dependent upon the symbiont producing light; i.e., the eyes showed no difference in gene expression between uncolonized animals and Δ*lux* mutant-colonized animals. Taken together, these data showed that the two remote tissues had different responses to light organ symbiosis, responses commensurate with their physiological functions. Immune function in the gills was reflected in a transcriptomic response to the presence or absence of the symbiont, with symbiont light production having no effect on gill transcriptomes. In contrast, modulation of light perception and response are the purview of the eyes, where the transcriptomic response to colonization of the light organ requires the production of light by the symbiont.

## AN UNDERSTANDING OF THE TRANSPORT OF CUES AND SIGNALS OFFERS A VAST HORIZON OF DISCOVERY

Although V. fischeri cells are extracellular symbionts, evidence that demonstrates abundant exchange of biomolecular cues and signals between the partners is accumulating.

**FIG 2:**
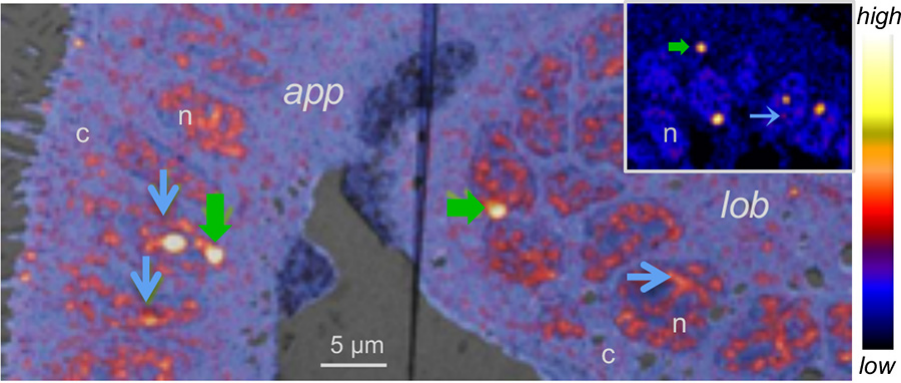
V. fischeri products, from intact cells or outer membrane vesicles (OMVs), taken up by host cells. Juvenile light organ visualized by nano-secondary ion mass spectrometry (nanoSIMS) after exposure to heavy-isotope-labeled V. fischeri cells or OMVs. In epithelial cells of both the appendage (*app*) and the main body of the organ (*lob*), labeling occurs predominantly in the nucleus (n), with vivid staining of the nucleoli (green arrows) and euchromatin (blue arrows). (Inset) With lower gain on the detector, the dominance of labeling in the nucleoli becomes highlighted. The color scale at right indicates labeling levels. c, cytoplasm.

As described above, studies of light organ colonization have revealed that host-symbiont communication occurs directly between the bacterial population and the crypt epithelium, as well as distant locations, affecting both the superficial regions of the juvenile light organ and other organs in the body. While an understanding of the modes by which these messages are delivered is in its infancy, nano-secondary ion mass spectrometry (nanoSIMS) has revealed that both the superficial ciliated epithelium and the crypt epithelium are unusually permissive to the uptake of symbiont-derived molecules (42) (Fig. 2). The transport of stable-isotope-labeled material into host cells can be observed after either colonization with labeled V. fischeri cells or exposure to outer membrane vesicles (OMVs) produced by these cells. The nanoSIMS signal is principally concentrated in the nuclei of those host cells in direct contact with the symbionts, particularly in the nucleolus and the euchromatin; nevertheless, the signal is also detected at lower levels in the cytoplasm. The onset of symbiosis greatly influences the expression of host genes, including protein-coding genes and microRNAs (30, 43). Whether the nuclear localization of the symbiont molecules has any part in these changes in host gene expression remains to be determined. Currently, the identification of the symbiont-derived molecules is the subject of intense investigation using targeted approaches, with particular interest in OMV cargo. For example, hybridization chain reaction fluorescence *in situ* hybridization (FISH) revealed high cytoplasmic levels of V. fischeri SsrA, which is carried in the OMVs (40). In addition to containing SsrA and other symbiont sRNAs, the OMVs contain proteins (20), some of which have predicted eukaryotic nuclear-localization signals. Studies of the host bloodstream revealed the presence of vesicles (likely OMVs) carrying bacterial sRNAs that have made their way into the circulatory system (40). How their presence in the circulatory system influences the host remains to be determined, but one might speculate that they are the vehicle by which the message of light organ colonization gets to the remote tissues, such as the eyes and gills (40). Clearly, explorations of these questions offer a broad frontier for this symbiotic system.

## FINAL CONSIDERATIONS—THE RICHNESS OF HOST-SYMBIONT COMMUNICATION DEEPENS

This contribution has focused on partner dialogue during the first hours to days of the squid-vibrio association. We did not cover several additional exciting arenas for future exploration, including communication between partners that is critical for the ongoing maturation of the relationship in the adult host, which occurs at 3 to 4 weeks following the colonization of hatchlings. In addition, while the majority of published work has been with a single strain of the symbiont, V. fischeri ES114 (44), recent reports have demonstrated an array of phenotypic differences in the symbiosis produced by different strains of V. fischeri (28, 29, 45, 46). These two major, but little-studied, areas of symbiosis promise to provide new insights into host-microbe communication.

While we have focused here on the onset of the squid-vibrio association, many exciting stories of signaling and cuing in symbiotic associations are emerging from the study of other systems. Such studies cover a broad array of topics, including the processes of nutrition, respiration, development, immunity, and metabolism (see, e.g., references [Bibr B47][Bibr B48][Bibr B52]); for current reviews of these phenomena in model systems of symbiosis, see references 53 and 54.
